# Expression of a Truncated Form of *ODAD1* Associated with an Unusually Mild Primary Ciliary Dyskinesia Phenotype

**DOI:** 10.3390/ijms23031753

**Published:** 2022-02-03

**Authors:** Lawrence E. Ostrowski, Weining Yin, Amanda J. Smith, Patrick R. Sears, Ximena M. Bustamante-Marin, Hong Dang, Friedhelm Hildebrandt, Leigh Anne Daniels, Nicole A. Capps, Kelli M. Sullivan, Margaret W. Leigh, Maimoona A. Zariwala, Michael R. Knowles

**Affiliations:** 1 Marsico Lung Institute/Cystic Fibrosis Research and Treatment Center, University of North Carolina at Chapel Hill, Chapel Hill, NC 27599, USA; weining_yin@med.unc.edu (W.Y.); amanda92@email.unc.edu (A.J.S.); patrick_sears@med.unc.edu (P.R.S.); xmbmarin@med.unc.edu (X.M.B.-M.); dangh@email.unc.edu (H.D.); leighanne_daniels@med.unc.edu (L.A.D.); nicole_capps@med.unc.edu (N.A.C.); kelli_sullivan@med.unc.edu (K.M.S.); margaret_leigh@med.unc.edu (M.W.L.); maimoona_zariwala@med.unc.edu (M.A.Z.); 2 Department of Pediatrics, School of Medicine, University of North Carolina at Chapel Hill, Chapel Hill, NC 27599, USA; 3 Department of Medicine, Boston Children’s Hospital, Harvard Medical School, Boston, MA 02113, USA; fhidbrant@harvard.edu; 4 Department of Pathology and Laboratory Medicine, School of Medicine, University of North Carolina at Chapel Hill, Chapel Hill, NC 27599, USA; 5 Department of Medicine, School of Medicine, University of North Carolina at Chapel Hill, Chapel Hill, NC 27599, USA

**Keywords:** *ODAD1*, *CCDC114*, cilia, primary ciliary dyskinesia, PCD, ciliopathy, outer dynein arm, docking complex

## Abstract

Primary ciliary dyskinesia (PCD) is a rare lung disease caused by mutations that impair the function of motile cilia, resulting in chronic upper and lower respiratory disease, reduced fertility, and a high prevalence of situs abnormalities. The disease is genetically and phenotypically heterogeneous, with causative mutations in > 50 genes identified, and clinical phenotypes ranging from mild to severe. Absence of *ODAD1* (*CCDC114*), a component of the outer dynein arm docking complex, results in a failure to assemble outer dynein arms (ODAs), mostly immotile cilia, and a typical PCD phenotype. We identified a female (now 34 years old) with an unusually mild clinical phenotype who has a homozygous non-canonical splice mutation (c.1502+5G>A) in *ODAD1*. To investigate the mechanism for the unusual phenotype, we performed molecular and functional studies of cultured nasal epithelial cells. We demonstrate that this splice mutation results in the expression of a truncated protein that is attached to the axoneme, indicating that the mutant protein retains partial function. This allows for the assembly of some ODAs and a significant level of ciliary activity that may result in the atypically mild clinical phenotype. The results also suggest that partial restoration of ciliary function by therapeutic agents could lead to significant improvement of disease symptoms.

## 1. Introduction

Primary ciliary dyskinesia (PCD) is a rare disease usually inherited in an autosomal recessive manner and characterized by chronic and recurrent lung infections leading to bronchiectasis, sinusitis, otitis media, reduced fertility, and laterality defects [1,2]. The predicted incidence of PCD is ~1:15,000, but the disease is under diagnosed and often misdiagnosed, in part due to the significant overlap of PCD symptoms with other more common diseases (e.g., asthma) [3]. PCD is caused by mutations that impair the biogenesis, structure, and function of motile cilia, including those in the upper and lower respiratory tract, eustachian tube, brain ventricles, oviduct, epididymis, and sperm flagella. In addition, mutations that affect ciliary proteins that are conserved in the embryonic nodal cilia result in an incidence of approximately 50% of situs inversus totalis and a significant incidence of other situs abnormalities (~10%) [4]. Much of the morbidity and mortality in PCD is caused by defective mucociliary clearance (MCC) in the airways, resulting in frequent pulmonary infections and the development of bronchiectasis [5]. A study comparing clinical features of bronchiectasis of PCD with bronchiectasis caused by other etiologies revealed that compared to alpha-1 antitrypsin deficiency (AATD), common variable immunodeficiency (CVI), and idiopathic bronchiectasis, subjects with PCD had worse lung function (forced expiratory volume in 1 s; FEV1) and developed disease at an earlier age [6]. Currently, there are no specific treatments for PCD.

Motile cilia are composed of several hundred proteins, and mutations in >50 different genes have been demonstrated to cause PCD [2,7]. Although all cases of PCD are caused by mutations that impair the biogenesis and/or function of motile cilia and result in defective MCC, the disease varies in severity from relatively mild to severe. Some of the variability in disease severity has recently begun to be explained by genotype/phenotype relationships. For example, mutations in *CCDC39* and *CCDC40* have been reported to cause PCD with a more severe clinical phenotype [8,9,10,11]. In contrast, PCD caused by mutation of the radial spoke protein, *RSPH1*, have been reported to have a mild clinical phenotype with a lower prevalence of neonatal respiratory distress and better lung function [12]. Cilia from mice and humans with *Rsph1*/*RSPH1* mutations beat in a clockwise rotational pattern, and we have reported that *Rsph1* knockout animals (*Rsph1*-/-) demonstrate a mild phenotype and a low level of mucociliary clearance [13]. This residual function likely explains the milder phenotype in humans, although the exact mechanism has yet to be identified. PCD is also heterogenous at the mutation level, with premature termination codons, missense, deletion, and splicing mutations all being reported as being causal, and this also likely contributes to the heterogeneous clinical phenotype [14,15].

Here we identified a subject with suspected PCD with situs inversus and an unusually mild clinical phenotype. Exome sequencing revealed biallelic splice mutations in *ODAD1* (NM_001364171.2; previously referred to as *CCDC114*) that encodes an approximately 79 kDa protein containing six coiled-coil domains (A0A6I8PTZ2). The Chlamydomonas orthologue of *ODAD1* (ODA1; DCC2) has been shown to be a component of the outer dynein arm docking complex (ODA-DC) that is essential for the assembly of the ODA [16]. Chlamydomonas *oda-1* mutants lack ODA in their flagella and swim with a slow, jerky manner. Similarly, mutations in human *ODAD1* typically result in the absence of ODA and almost complete ciliary immotility, and result in a typical PCD clinical phenotype, including a high incidence of neonatal respiratory distress, low levels of nasal nitric oxide, sinusitis, otitis media, bronchiectasis, and situs inversus totalis [17,18].

To elucidate the mechanism responsible for the mild phenotype in this subject, we cultured nasal epithelial cells at the air–liquid interface and performed molecular, biochemical, and functional assays. The results show that the non-canonical splice mutation results in the production of a truncated protein that is assembled into the axoneme and allows for the attachment of some dynein arms and a significant amount of ciliary activity that is likely responsible for the mild phenotype. Importantly, the mild phenotype of this subject demonstrates that treatments that restore even partial ciliary function may provide significant improvement in the clinical phenotype for patients with more severe forms of PCD.

## 2. Results

### 2.1. Patient Characteristics and Mutation Identification

**Figure 1:**
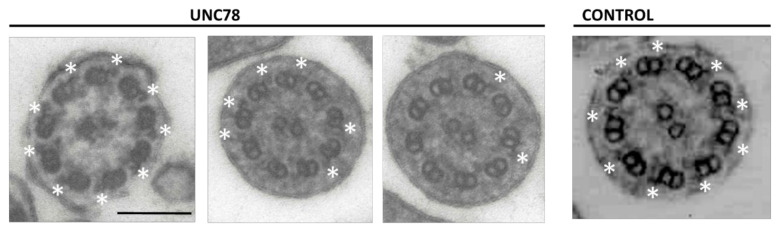
Transmission electron micrographs (TEM) of nasal cilia from subject UNC78 show the presence of normal outer dynein arms (ODA; white asterisks) on multiple cilia. While many ciliary cross-sections showed no ODA, some sections had a completely normal complement of nine. UNC78 (left panels) show 3 examples from subject UNC78; Control (right panel) shows a normal ciliary cross-section. Scale bar = 0.15 μm.

The subject (UNC78) has situs inversus totalis but did not have neonatal respiratory distress. She developed a wet productive cough as a young child and had several ear infections, but has had no ear infections since her teenage years, and participated in athletics at a competitive level. Based largely on the presence of situs inversus, a frequent productive cough, and a few episodes of bronchitis, she was referred for evaluation and PCD was suspected. At age 16, she had a normal chest CT scan (except for dextrocardia), normal spirometry and pulmonary exercise study, and normal sinus imaging. A workup for cystic fibrosis (sweat Cl^−^ and genetic tests for 14 CFTR mutations) was negative. Examination of transmission electron micrographs (TEM) obtained from a nasal biopsy revealed a reduction in the number of outer dynein arms (ODA), with most cilia having no clearly discernable dynein arms. However, a number of axonemes had several apparently normal ODA structures (Figure 1; see below). Because ODA defects are a frequent cause of PCD, we screened 18 PCD-related genes to identify the cause of this individual’s disease.

Genomic DNA was extracted from whole blood and analyzed using a high-throughput exon-sequencing technique (Access Array Microfluidic technology; Fluidigm, South San Francisco, CA, USA) as previously described [19,20]. This analysis identified a previously reported homozygous splice mutation (c.1502+5G>A (pSer469Argfs*7)) in IVS14 of *ODAD1* [17]. Analysis of parental DNA confirmed an autosomal recessive pattern of inheritance, and no other pathogenic mutations in known PCD genes that were screened were identified. These data support the conclusion that the splice mutation in *ODAD1* was the cause of the patient’s disease symptoms, as a result of the defective dynein arms. Mutations in *ODAD1* have previously been reported as a rare cause of PCD, occurring in < 2% of reported cases [2]. However, the subject’s clinical phenotype differed in several ways from other PCD subjects with previously identified mutations in *ODAD1* (Table 1 and [17,18]). Specifically, the subject had nasal nitric oxide (nNO) levels of 188 and 205 nL/min, above the PCD specific value of < 77 nL/min [21]. The subject also had no history of neonatal respiratory disease, which is reported to occur in over 75% of PCD subjects [22]. At age 34, the subject had a normal FEV_1_ value (95% predicted), no evidence of bronchiectasis on a chest CT scan, and no chronic/recurrent sputum cultures for typical bacterial pathogens [1,2,3]. As shown in Table 1, these features are clearly different than what has been observed in other PCD patients with identified mutations in *ODAD1*, with all other subjects having low levels of nNO and recurrent otitis media, and most having neonatal respiratory distress, bronchiectasis, sinusitis, respiratory bacterial pathogens, and low FEV_1_ (% predicted).

### 2.2. Quantification of ODA in ODAD1 Subjects

We previously reported the c.1502+5G>A (previously identified as c.1391+5G>A) variant in a heterozygous state in a PCD subject (UNC897; Table 1; [17]), and showed that the mutation resulted in the skipping of exon 14 and the introduction of a premature translation codon (pSer469Argfs*7). Interestingly, TEMs from subject UNC897 (who was 3 years old at the time of diagnosis) revealed a similar pattern as observed for subject UNC78, with most axonemes showing no clear ODA, while multiple axonemes had five or more ODA (Table 2). In addition, this subject had no evidence of bronchiectasis and an FEV_1_ of 94% at age 10. Examination of nasal cilia from two subjects homozygous for the more common splice variant, c.853G>A (previously reported as c.742G>A), that occurs in the last base of exon 9, also showed a small number of ODA, although a subject with compound heterozygous variants (UNC891) had no visible ODA. For comparison, we also quantified ODA in a nasal biopsy from subject UNC961, who has homozygous stop mutations (c.448C>T; p.Arg150*) in ODAD1, and observed no clear ODA. The presence of normal ODA in subjects with splice mutations indicates that there is some level of normal splicing (leakiness) occurring, or that a truncated protein with partial function is being expressed.

### 2.3. Ciliary Activity of c.1502+5G>A Mutant Cells in Vitro

**Figure 2:**
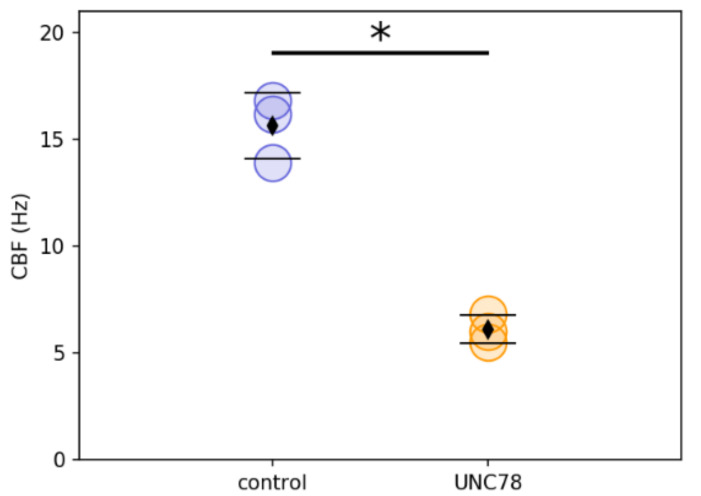
Ciliary beat frequency in ALI cultures of healthy control HNE cells and UNC78 HNE cells. Each point represents the average of 3–4 cultures from a separate experiment. Diamond symbol, the mean; error bars, std dev.; *, *p* < 0.001 by Student’s *t*-test. Blue symbols = control; orange symbols = UNC78.

To investigate possible mechanisms for the atypically mild phenotype in UNC78, we obtained nasal epithelial cells, expanded them in culture, and allowed them to differentiate in air–liquid interface (ALI) cultures. Ciliary activity was easily visible in cultures from subject UNC78, with many areas of the cultures showing patches of actively beating ciliated cells (Videos S1 and S2). These results differ from previous studies that reported almost complete ciliary immotility in subjects with mutations in *ODAD1* [17,18]. However, the beat frequency was 6.1 +/− 0.6 Hz which is significantly reduced compared to control cells (15.6 +/− 1.5 Hz; *p* < 0.001; Figure 2).

Examining ciliary waveform by tracing the x–y (top–down) motion of individual cilia showed a mostly planar beat in UNC78 cultures, similar to control cultures. Examination in the x–z plane (profile) by tracing the motion of individual cilia and measuring the angle of the end recovery and end effective position appeared to show a shortened recovery stroke in the UNC78 cells, although this difference was not significant. (Videos S3 and S4; Figure S1). Thus although the CBF was significantly reduced, the cilia appeared to beat with a mostly normal waveform and in a coordinated fashion, potentially providing sufficient force for some level of MCC.

### 2.4. RNA Expression Analysis

**Figure 3:**
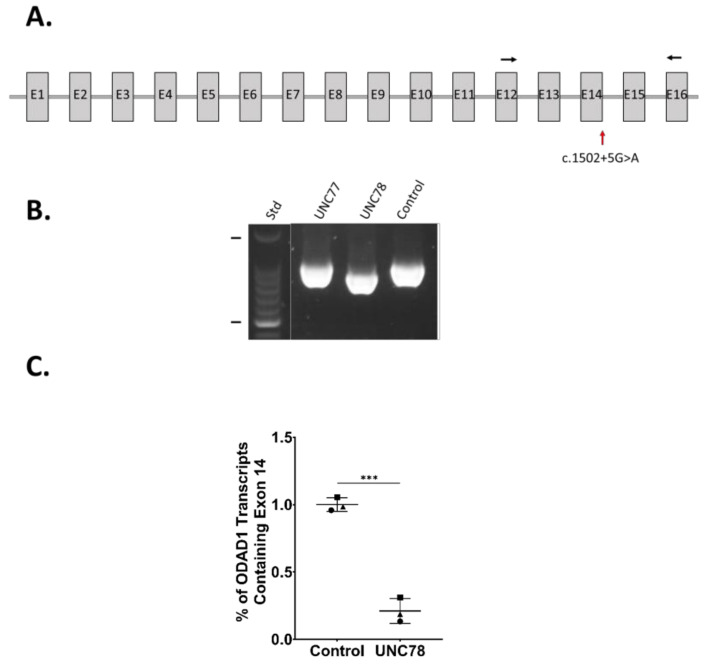
(**A**) Schematic of the *ODAD1* transcript (NM_001364171.2) showing the relative locations of the c.1502+5G>A variant (red arrow) and the primers used for RT-PCR (black arrows). (**B**) The major product produced by RT-PCR of UNC78 was shorter than that produced from UNC77 and an unrelated control, due to the skipping of exon 14. (**C**) ddPCR demonstrated the expression of a greatly reduced level of *ODAD1* transcripts containing exon 14 in samples from UNC78. Symbols, datapoints from individual experiments; lines, the mean and std. dev.; ***, *p* < 0.001 by a two-tailed *t*-test. (*n* = 3 experiments).

The abundant ciliary activity along with the presence of some ODA (as evidenced by TEM, Figure 1) suggested that the *c.1502+5G>A* variant might be leaky, allowing some level of normal splicing to occur that would encode the full-length ODA1 protein and result in the assembly of some complete ODAs. Alternatively, the skipping of exon 14 is predicted to lead to an mRNA transcript encoding a truncated protein lacking exons 14, 15, and 16. RT-PCR using primers that spanned exon 14 (Table S1) showed that the major product from the subject was shorter than in the controls, and sequencing confirmed the deletion of exon 14 (Figure 3A,B), as we previously reported for subject UNC897, who is heterozygous for the *c.1502+5G>A* variant [17]. To further examine the possibility of some normal splicing, we performed quantitative ddPCR on RNA isolated from differentiated cultures of nasal epithelial cells from UNC78 and control human nasal epithelial (HNE) cells. While the expression of *DNAI1* was similar between the samples (control, ~450 copies/μL; UNC78, ~520 copies/μL; *n* = 3), the level of *ODAD1* transcripts containing exon 14 was reduced to 21% in UNC78 (Figure 3C).

**Figure 4:**
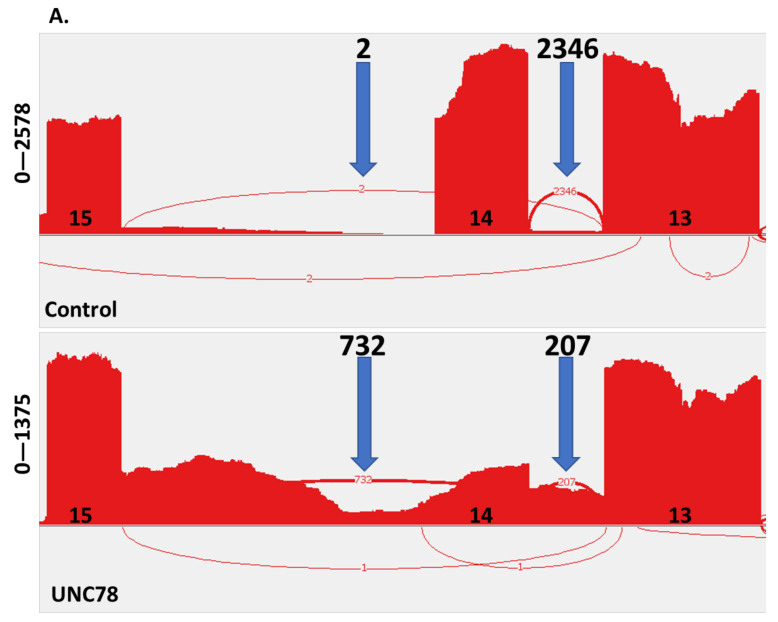

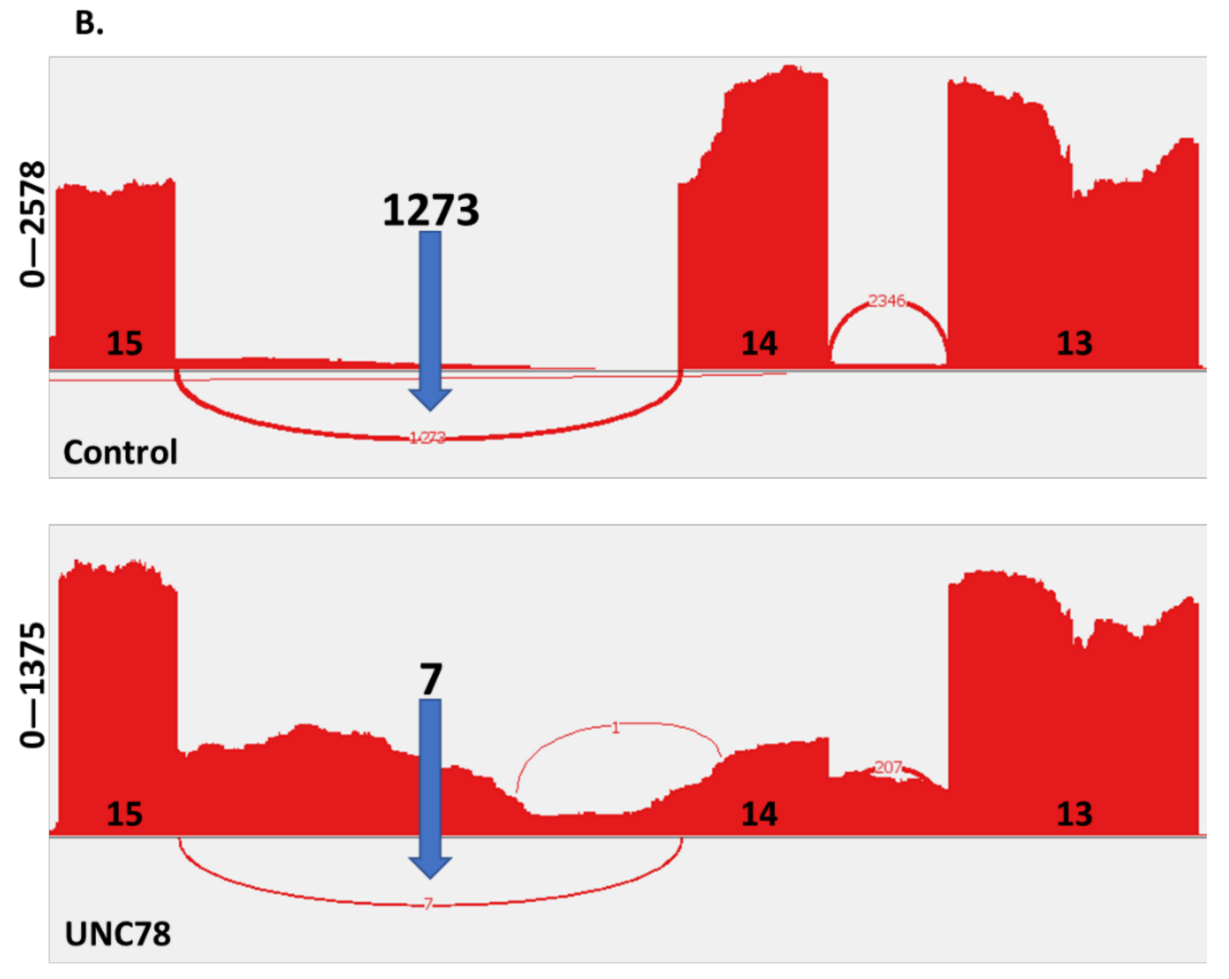
Analysis of *ODAD1* transcripts. RNA-seq was performed on RNA isolated from differentiated cultures of control HNE cells and UNC78. (**A**) Sashimi plot showing splicing from exon 13 in control (top) and UNC78 (bottom) samples. (**B**) Sashimi plot showing splicing from exon 14. Location of exons (13, 14, 15) is shown along the x-axis; y-axis indicates number of reads. Blue arrows, number of spliced transcripts between the indicated exons. The sample from UNC78 has little evidence of correct splicing. See text for details.

To further examine the defect caused by the *c.1502+5G>A* variant, RNA-seq was performed on RNA isolated from differentiated cultures of UNC78 and controls. This analysis confirmed the deletion of exon 14 in the majority of *ODAD1* transcripts from UNC78 (732/940; ~78%; Figure 4A). Only 207 of the 940 transcripts identified correctly joined exon 13 to 14 in UNC78, compared to 2346 of 2348 (>99%) in the control. However, there were very few (seven) transcripts identified that showed a correct splice from exon 14 to exon 15 in UNC78, compared to 1273 transcripts in the control sample (Figure 4B). Splicing of upstream exons (i.e., 6–7) were similar between UNC78 and the control. Therefore, although these studies show that some of the mis-spliced *ODAD1* transcripts in the c.1502+5G>A mutant cells contain exon 14, these will not encode the full-length protein, and are unlikely to account for the level of ciliary activity observed.

Interestingly, the RNA-seq data also revealed that the majority of *ODAD1* transcripts encode a longer isoform of the protein than previously reported (A0A6I8PTZ2). This sequence is in frame with the 670 amino acid protein previously reported in UniProt (Q96M63; NM_144577.4) but contains an additional 37 amino acids at the amino terminus and has a predicted molecular weight of 79,035 compared to 75,046 for the shorter isoform. Identification of the longer isoform of *ODAD1* has implications for clinical labs offering *ODAD1* testing. The addition of 37 amino acids in the coding region will change the variant nomenclature. Importantly, the additional 37 amino acids in the coding region of the long isoform will need to be included in mutation profiling and sequence analysis, as these were previously not likely ascertained due to their presumed presence in the 5′UTR.

### 2.5. Expression of a Truncated Form of ODAD1

The c.1502+5G>A mutation results in the deletion of exon 14 and is predicted to result in the expression of a truncated *ODAD1* protein. To determine if this isoform was stably expressed and may retain partial function, we examined ciliary isolates by Western blotting. Two different antibody preparations recognizing different epitopes of the ODAD1 protein were first characterized on preparations of control cilia and lysates from HEK293 cells expressing an HA-tagged *ODAD1* cDNA coding for the 670aa isoform (Q96M63) or a shorter cDNA coding for the predicted truncated isoform of *ODAD1* (437aa). Antibody HPA042524 targets a 90 aa sequence from the mid-region of *ODAD1* (Table S3) and detects both the full-length and truncated version of *ODAD1* expressed in 293 cells, and a slightly larger protein in isolated cilia (Figure S2). In contrast, antibody PAS-46305, that targets the carboxy terminal 50 aa (Table S3), only detects the full-length *ODAD1* products, and not the product from the truncated cDNA (Figure S2).

**Figure 5:**
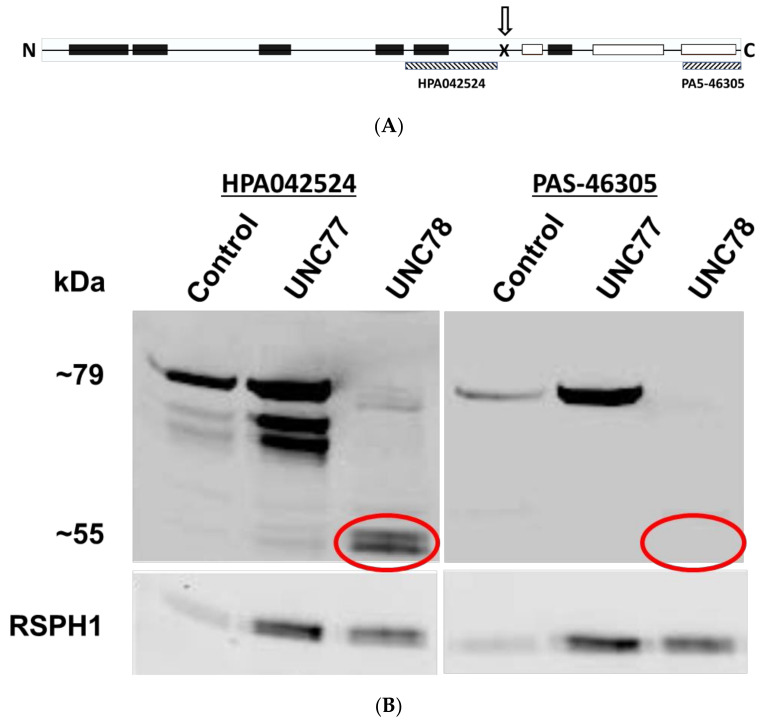
Western blotting of axonemal proteins. (**A**) Diagram showing the approximate location of the pSer469Argfs*7 mutation (arrow, X) and the antigenic site of the two antibodies used (hatched boxes). Predicted coiled–coil domains are shown in black boxes; disordered domains are shown as white boxes. (**B**) Cilia were isolated from ALI cultures of control, UNC77, and UNC78 cells and analyzed by Western blotting using two different *ODAD1* antisera. Antisera against the mid-region of *ODAD1* recognizes two truncated proteins in the sample from UNC78 (left), while the antisera against the C-terminus does not recognize these proteins (right).

These antibodies were used to analyze ciliary axonemes isolated from control (normal human bronchial epithelial (HBE) cells), UNC77 (HNE from a parent of UNC78; obligate heterozygote for c.1502+5G>A), and UNC78 cells. As shown in Figure 5, antibody HPA042524 recognizes the full-length 79 kDa protein in the control and UNC77 lysates. Variable amounts of two shorter proteins were also detected in some preparations; we suspect these are degradation products of the full-length isoform. However, in cilia from the UNC78 cells, a doublet of two smaller proteins of approximately 55 kd are detected. When probed with the antibody raised against the carboxy terminus (PAS-46305), only the full length *ODAD1* protein is detected in the ciliary axonemes isolated from control and UNC77 cells, while no reactive proteins are detected in the ciliary axonemes from subject UNC78. Normalization to the *RSPH1* signal (as a marker of ciliary proteins) indicates that the level of truncated *ODAD1* is approximately 11.2% of the level of full-length protein in UNC 77 (13.5%, 8.8%; *n* = 2). Probing of replicate blots for *DNAI1*, a component of the ODA, was also positive in ciliary axonemes from UNC78, providing further evidence for the assembly of some complete ODA (Figure S3). These results demonstrate that the c.1502+5G>A mutation results in the production of a truncated *ODAD1* protein that is incorporated into the ciliary axoneme and retains partial function.

### 2.6. Immunofluorescence Localizes Truncated ODAD1 to Cilia

**Figure 6:**
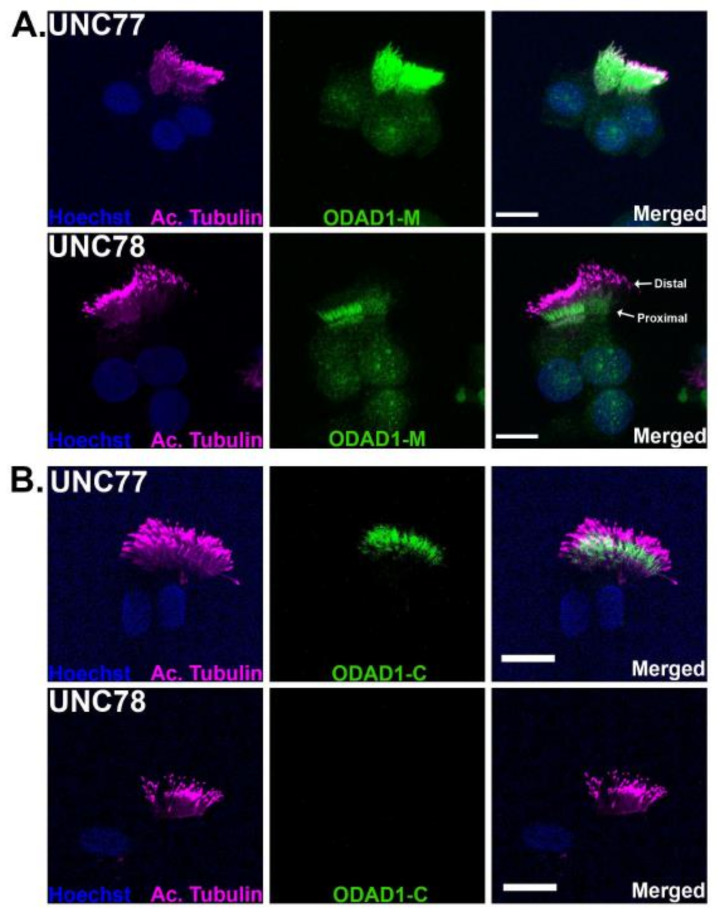
Immunostaining of *ODAD1*. (**A**) Immunofluorescent staining of UNC77 with the antisera against the mid-region of *ODAD1* shows strong positive staining throughout the cilia. In contrast, staining of UNC78 shows a weaker signal, mostly localized to the proximal region of the axoneme. (**B**) Staining with the antisera against the C-terminal region of *ODAD1* shows a clear signal in the UNC77 cells, but no detectable staining in UNC78. Cilia were labeled with an antibody against acetylated tubulin. These results confirm the presence of truncated *ODAD1* in the axonemes. Scale bars = 10 μm. (*n* = 2–3 experiments).

To confirm the incorporation of the truncated *ODAD1* protein into the ciliary axonemes of subject UNC78, ciliated cells from cultures of UNC77 and UNC78 were immunostained with antisera against the mid- and C-terminal region characterized above (antibodies HPA042524 and PAS-46305). Cells were also stained with an antibody against acetylated tubulin to label cilia. Cilia from the control (UNC77) cultures were labeled along the entire length of the ciliary axoneme (Figure 6) with antibody HPA042524 against the mid-region of *ODAD1*, as expected for an ODA protein. In contrast, ciliated cells from UNC78 showed a greatly reduced level of staining with HPA042524, consistent with the reduced number of ODA and the reduced level of *ODAD1* protein detected by Western blotting. The staining appeared localized to the proximal region of the ciliary axoneme. Staining with the antibody against the C-terminus of *ODAD1* (PAS-46305), appeared to preferentially label the proximal region of cilia in control cells, but was undetectable in the cilia from UNC78. This result agrees with the Western blotting that demonstrated the absence of any full-length *ODAD1* in the cilia from UNC78 cells, and confirms that the signal observed is derived from the truncated form of *ODAD1*.

## 3. Discussion

Individuals with PCD suffer from a variety of symptoms including recurrent lung, sinus and ear infections that are the result of defective MCC, due to mutations that impair the biogenesis and/or function of motile cilia. The symptoms vary in severity among subjects, and recent studies have begun to explore the basis for this heterogeneity. At the genetic level, it is clear that mutations in some genes (e.g., *RSPH1* [12]) are associated with a milder phenotype, while other genes (e.g., *CCDC39*, *CCDC40* [8]) are associated with a more severe clinical phenotype. Additionally, some genes have a more critical role in some tissues, so that mutations in these genes may result in variations in phenotype. For example, while most PCD causing mutations result in male infertility, subjects with mutations in *RSPH4A* have been reported to be fertile [23]. Mutations in proteins (e.g., *HYDIN, RSPH1*) that affect the central pair, which are not present in nodal cilia, cause PCD without causing the situs abnormalities associated with PCD (e.g., situs inversus, situs ambiguous) [4]. Also contributing to the heterogeneity of disease is the wide spectrum of the types of identified mutations, with many different mutations occurring in the same gene. For example, over 80 different PCD causing mutations have been identified in *DNAH5* (https://medlineplus.gov/genetics/gene/dnah5/#conditions) (accessed on 2 February 2022). Finally, it is likely that other factors, including other genetic factors, environmental factors, age at diagnosis, and treatment regimes, will all influence disease severity and progression.

*ODAD1*, also known as *CCDC114*, is an essential component of the outer dynein arm-docking complex required for the correct attachment of the ODA to the microtubule doublet. Studies in Chlamydomonas have shown that absence of the *ODAD1* orthologue, DCC2, results in compete absence of the ODA and impaired swimming ability [16]. Similarly, cilia from PCD patients with mutations in *ODAD1* show a reduction of ODA and were mostly observed to be immotile, with only a few cilia showing a stiff, dyskinetic beating [17,18].

In contrast, differentiated cultures from subject UNC78 showed many areas of highly active ciliary activity. Examination of individual ciliated cells by high-speed video microscopy revealed that ciliary activity was abundant and ciliary waveform maintained a mostly normal, planar waveform, although CBF was reduced by approximately 50%. The reduction in CBF would likely result in a reduced rate of MCC [24]. However, in spite of the ciliary defect, this patient was noteworthy because of the extremely mild nature of her disease. In particular, although essentially all PCD patients >18 years of age have clear-cut bronchiectasis, subject UNC78 had no bronchiectasis on a chest CT scan at age 34.

Our studies provided the following evidence that the higher than expected level of ciliary activity and the mild phenotype are the result of the expression of a truncated isoform of *ODAD1* that retains partial function. First, analysis of ciliary ultrastructure by TEM showed the clear presence of intact ODA. In fact, several TEM images of ciliary cross-sections from subject UNC78 appeared to have a complete complement of nine ODA. This is in clear contrast to what was observed from a subject with two nonsense mutations in *ODAD1* that showed a complete absence of ODA. Data from Chlamydomonas also demonstrated that the absence of DCC2 also results in a complete lack of ODA. Interestingly, while some cross-sections had many ODA visible by TEM, the majority of ciliary cross-sections examined (~75%) had none. This pattern of occasional cross-sections showing multiple ODAs was also observed in other subjects with splice mutations, although it is not clear if these arise from a low level of normal splicing or the production of a partially functional protein. Immunofluorescence studies of UNC78 revealed that the truncated *ODAD1* protein appeared to localize to the proximal region, suggesting that the cross-sections with no ODA may have been derived from the more distal portion of the cilia. Why the truncated form of *ODAD1* would preferentially localize to the proximal region is unknown at present, and further studies will be required to understand the mechanism.

Second, cultured ciliated cells from UNC78 showed large areas of active cilia, with a near normal waveform. This result is also not consistent with a complete loss of ODA nor with the previous reports of immotile or mostly immotile cilia in other subjects with different mutations in *ODAD1*.

Third, RNA analysis shows the majority of the *ODAD1* transcripts present in UNC78 cells are lacking exon 14, with little evidence for the production of a normal, full-length transcript.

Fourth, immunofluorescence of ciliated cells with antisera that recognize a mid-region epitope showed positive staining for *ODAD1* in cilia from UNC78, although it was reduced compared to control cilia. However, there was no detectable staining with an antibody against the C-terminal region of *ODAD1*, again suggesting that little or no full-length *ODAD1* protein was being produced. Similarly, Western blotting of isolated cilia from the subject showed the presence of two shorter proteins, approximately the size of the predicted truncated form of *ODAD1*. These proteins reacted with antibodies recognizing the upstream region of *ODAD1*, but did not react with the C-terminal antibody. This is also consistent with the expression of the truncated form of *ODAD1*. These smaller isoforms were enriched in the ciliary axoneme compared to the cell lysate, indicating that they were a stable component of the axoneme (data not shown). In addition, there was no full-length *ODAD1* protein detected in the ciliary lysate using either of the antisera tested.

Together, these results strongly suggest that the truncated version of *ODAD1*, caused by the splicing mutation (c.1502+5G>A (pSer469Argfs*7)) and missing exon 14, retains partial function and is responsible for the assembly of some ODA. *ODAD1* is predicted to contain multiple coiled–coil domains that are likely important for interaction with other ODA-DC proteins, including *CCDC151* [25]. Importantly, the truncated protein produced by the c.1502+5G>A mutation would retain most of the predicted coiled–coil domains (5/6), while the disordered C-terminal region would be lost. Although the level of ODA is reduced compared to normal, it is clearly sufficient to generate substantial ciliary activity. Further, it seems likely that the remaining ciliary activity is at least partially responsible for this subject’s mild disease. What remains unknown is whether the ciliary activity is contributing directly to mucociliary clearance, or whether the ciliary activity is mitigating disease symptoms by other mechanisms. For example, it has been reported that beating cilia can sense and respond to stress by increasing hydration of mucus (through the release of ATP) [26]. By regulating the hydration or other properties of the mucus layer, the ciliated cell may improve the ability of a PCD subject to clear mucus by cough, thus improving the clinical phenotype in the absence of MCC. Alternatively, it has also been reported that ciliary activity stimulates the release of nitric oxide, and the levels of nasal nitric oxide in UNC78 are indeed much higher than in other PCD patients, as shown in Table 1. Nitric oxide plays several roles in the airways, including functioning as an antimicrobial agent and as a bronchodilator, both of which could reduce the severity of disease in PCD patients [27]. Additional studies in a larger number of patients, including direct measurements of MCC in PCD subjects with different genotypes, will be required to distinguish between these and other possibilities.

Finally, these studies provide insight into the development of therapies for this rare disease, for which there are no specific treatments available. While the ideal therapy would restore normal ciliary activity to every ciliated cell and result in the full restoration of MCC, this is not yet possible. However multiple approaches are currently being developed, including CRISPR based genetic approaches, RNA based replacement therapies, read-through agents, and molecules designed to correct splicing defects. The mild disease phenotype observed in UNC78 suggests that even partial correction of ciliary activity may lead to improved outcomes, although the level and distribution of ciliary activity that is required for meaningful clinical improvement is unknown.

## 4. Materials and Methods

### 4.1. Human Subjects

Studies involving human tissues were approved by the Institutional Review Board at the University of North Carolina (protocol #98-1015; initially approved 3 June 1998) and were performed in compliance with all ethical regulations. Written informed consent was acquired from the PCD-affected individuals, their parents, and other participants. Demographic and clinical data were obtained by standardized methodologies as previously reported [17].

### 4.2. Identification of Genetic Variant

Genomic DNA was extracted from whole blood for UNC78 and UNC961. Mutation analysis was performed, using high-throughput exon-sequencing on Illumina^®^ Highseq 2000 (Illumina Inc, San Diego, CA, USA) after barcoded multiplex PCR on a Fluidigm 48-Access Arrays TM Microfluidic system (Fluidigm, South San Francisco, CA, USA) as previously described [19,20]. A total of 18 PCD-related genes were screened (*CCDC103, CCDC39, CCDC40, DNAAF1, DNAAF2, DNAAF3, DNAH11, DNAH5, DNAI1, DNAI2*, *DNAL1, NME8, ODAD1 (CCDC114), OFD1, RPGR, RSPH4A, RSPH9, SPAG1*). Segregation analysis was carried out using Sanger sequencing as previously described [17].

### 4.3. Enumeration of ODA

Transmission electron micrographs of cilia in cross-section were obtained from nasal biopsies and examined for the presence of ODA [28]. Briefly, an experienced investigator (MRK) evaluated individual cilia to determine if the image was of sufficient quality to be counted, based on the appearance of clearly resolved microtubule doublets and central pair structures. The number of normal ODAs were counted and recorded. Results were reviewed and confirmed by two additional investigators (MWL, LEO).

### 4.4. Expansion and Culture of Airway Epithelial Cells

Nasal epithelial cells were obtained from the PCD subject (UNC78), an obligate heterozygote (parent; UNC77), and healthy donor controls by means of a nasal scrape and expanded using conditional reprograming techniques [29,30]. Expanded cells were then plated on collagen coated Millicell inserts and cultured at the air/liquid interface (ALI) until ciliary differentiation occurred. Initial studies were performed using UNC-ALI media, while later studies were performed using Pneumacult-ALI (Stemcell Technologies, Cambridge, MA, USA). Human bronchial epithelial (HBE) cells (passage 1) were obtained from the UNC Cell and Tissue Culture Core under protocols approved by the University of North Carolina Institutional Review Board. Normal HBE cells obtained from non-smoking male and female donors were cultured at an air/liquid interphase (ALI) as previously described and cultured as previously published in detail in UNC-ALI media [31].

### 4.5. Measurement of Ciliary Activity

Ciliary beat frequency (CBF) was measured as previously described [13,29]. Briefly, differentiated cultures were washed quickly with PBS to remove debris and cultures were placed in a temperature-controlled stage top incubator maintained at 37 °C via a Tokai HIT controller (model INU-TIZ-F1) and a microscope stage-heater block. Videos were recorded from 10–12 equally spaced fields across the culture surface using a Nikon Eclipse TE2000 microscope (Nikon Instruments Inc, Melville, NY, USA) and Basler acA1300–200 um camera controlled by SAVA software (version 2.0.8W, Ammons Engineering, Clio, MI, USA) and analyzed using SAVA whole field analysis. For evaluation of the waveform and beat direction, high-resolution videos of isolated ciliated cells were recorded at 200 fps with a 60× Plan-Apo oil objective lens (NA = 1.4), DIC optics, and ambient temperature. Analysis of videos was conducted by investigators blinded to the cells’ genotype. Videos were replayed in slow motion (1/7 of the true speed). For waveform analysis, three cilia were traced manually at the end recovery and end effective positions in four ciliated cells from each genotype. To evaluate the direction of the ciliary beat, the tip positions of four to five individual cilia were manually traced, and the orientation of the ciliary beat was represented in a two-dimensional plot.

### 4.6. RNA Analysis

RT-PCR: RNA was isolated from cultures of airway epithelial cells using a RNeasy kit (QIAGEN, Germantown, MD, USA) according to the manufacturer’s protocols. First-strand cDNA was synthesized with SuperScript II Reverse Transcriptase (Thermo Fisher, Waltham, MA, USA, cat.# 18064014). PCR was performed with Apex Taq RED master mix (Genesee Scientific, San Diego, CA, USA) according to the manufacturer’s protocols. Primers and annealing temperatures are listed in Table S1.

ddPCR: Droplet digital PCR (ddPCR) was performed on cDNA synthesized from isolated RNA from cultured nasal epithelial cells. Using the Bio-Rad QX200 Automated Droplet Generator, at least 10,000 droplets were generated from 20 uL ddPCR reaction mixes consisting of 250 nM primers, 900 nM probes, and 1X ddPCR Supermix for Probes (No dUTP) (Bio-Rad, Hercules, CA, USA). PCR reactions were performed according to cycling conditions listed in the manufacturer’s protocols. Using the Bio-Rad QX200 Droplet Reader, the fluorescent positive and negative droplets were then counted. *ODAD1* probes were fluorescently labelled with FAM, while the reference gene, *DNAI1*, probe was labelled with HEX. After normalization to the concentration of the reference gene, *DNAI1*, *ODAD1* transcript concentrations were calculated using Quantasoft software (version 1.0.596, Bio-Rad, Hercules, CA, USA). Quantification of the levels of full-length *ODAD1* (transcripts containing exon 14) was calculated by dividing the concentration of normalized *ODAD1* exons 13–14 transcripts by the concentration of normalized *ODAD1* exons 10–11. Primer/probe assays for *ODAD1* exons 10–11 and *ODAD1* exons 13–14 were generated using Primer3Plus and custom ordered from Bio-Rad. PCR using the same reagents and cycling conditions on undifferentiated and differentiated human bronchial epithelial cells cDNA samples followed by sequencing of the amplicons was performed to validate these custom assays. All primer/probe assay sequences are listed in Table S2.

RNA-seq: Paired-end RNA-seq of 150 bp was performed with Illumina (standard RNAseq, Genewiz Inc, South Plainfield, NJ, USA) and yielded 100 million reads per sample. Raw sequences in FASTQ format were mapped to the GRCh38 reference genome and Gencode v37 gene, and transcript annotations using the STAR aligner [32], resulting in alignment BAM files. RNA-seq alignments in BAM format were visualized using the IGV browser [33], and splice patterns interrogated with a Sashimi plot.

### 4.7. Western Blotting

Western blotting of ciliary proteins was performed using standard protocols, as previously described [7,34]. For characterization of anti-*ODAD1* antibodies, plasmids encoding the 670 amino acid isoform (Uniprot.org, accession # Q96M63) and the predicted 437 amino acid truncated isoform were constructed by GeneScript (GeneScript, Piscataway, NJ, USA). Both constructs contained an amino-terminal HA-tag. Plasmids were transfected into HEK293 cells using Lipofectamine 2000 (Invitrogen, Carlsbad, CA, USA) and cell lysates were prepared in RIPA buffer. Cilia from differentiated cultures of airway epithelial cells were isolated as previously described [7,34], pelleted, and resuspended in 1× LDS (Invitrogen, Waltham, MA, USA) lysis buffer. Proteins were separated on 4–12% Bis-Tris gels (Invitrogen, Carlsbad, CA, USA), transferred to nitrocellulose membranes and blocked in OneBlock (Genesee Scientific, San Diego, CA, USA). Membranes were probed with the antibodies and concentrations listed in Table S3 and visualized using a LI-COR Odyssey Scanner (LI-COR Biotechnology, Lincoln, NE, USA).

### 4.8. Immunofluorescence

Immunofluorescent staining of isolated HNE cells was performed using previously described protocols [25]. Isolated cells from HNE cell cultures in 1X PBS were allowed to settle on 1% Alcian Blue (Sigma-Aldrich Co, St. Louis, MO, Il, USA)-treated slides/cover slips for 20 min. on ice. Cells were fixed with 2% paraformaldehyde for 10 min. and washed with 1X PBS three times. Cells were permeabilized with 0.2% Triton X-100 in PBS for 15 min. Cells were incubated in blocking solution (5% donkey serum, 3% BSA, 0.1% Triton X-100 in 1X PBS) for 1 h at RT., followed by incubation in primary antibody diluted in blocking solution either for 1 h at RT or overnight at 4 °C. After washing with 1X PBS three times, cells were incubated in secondary antibody solutions for 2 h at RT. After washing with 1X PBS four times, slides were mounted with ProLong Diamond Antifade Reagent (Thermo Fisher, Waltham, MA, USA). Nuclei were stained using DNA dye Hoechst33342 (Invitrogen, Waltham, MA, USA). Slides were imaged with a Zeiss 800 upright confocal microscope with a 63X/1.4 oil objective. No detectable staining was observed for isotype-matched antibody or no primary antibody control slides. Using FIJI software (version 2.1.1), all images were processed, pseudo-colored, and brightness/contrast was adjusted identically across subjects.

## Figures and Tables

**Table 1:** Demographic, Clinical, and *ODAD1* Mutations in PCD-Affected Individuals.

													Allele 1			Allele 2		
UNC #	Study	Sex	Age	Ethnicity	nNO nl/min ^a^	Situs Status	Neo RDS	Bxsis	Sinusitis	Otitis Media	Pathogens ^b^	FEV_1_ % Pred	Exon/Intron	Base Change	Amino Acid Change	Exon/Intron	Base Change	Amino Acid Change
Homozygous Mutations																		
78	Current	F	34	white ^c^	205/188 ^d^	SI	no	no	no	no	none	95 ^e^	Int 14	c.1502+5G>A	p.Ser469Argfs*7	Int 14	c.1502+5G>A	p.Ser469Argfs*7
359	ref [17]	F	39	white	36.0	SS	yes	yes	yes	yes	Ps a	84	Ex 9	c.853G>A ^f^	p.Ala285Serfs*52	Ex 9	c.853G>A ^f^	p.Ala285Serfs*52
360	ref [17]	F	33	white	31.5	SS	yes	yes	yes	yes	Ps a; St p	92	Ex 9	c.853G>A ^f^	p.Ala285Serfs*52	Ex 9	c.853G>A ^f^	p.Ala285Serfs*52
961	Current	F	18	Asian/Indian	18.9	SI	no	yes	yes	yes	Ps a	42	Ex 6	c.448C>T	p.Arg150*	Ex 6	c.448C>T	p.Arg150*
Comound Heterozygous Mutations																		
891	ref [17]	F	59	white	32.4	SS	yes	yes	yes	yes	Ps a	57	Ex 9	c.853G>A ^f^	p.Ala285Serfs*52	Int 7	c.598-2A>G	p.Glu200Glyfs*60, p.Glu200_Val221delins ^g^
897	ref [17]	M	10	white	9.6	SS	yes	no	no	yes	none	94	Int 14	c.1502+5G>A	p.Ser469Argfs*7	Ex 9	c.853G>A ^f^	p.Ala285Serfs*52
1107	ref [17]	F	34	white	6	SS	no	yes	yes	yes	Ps a; Kl p	46	Ex 9	c.853G>A ^f^	p.Ala285Serfs*52	Ex 11	c.1050delT	p.His350Glnfs*14

Abbreviations: M, male; F, female; Neo RDS, neonatal respiratory distress in full-term birth; Bxsis, bronchiectasis; nNO, nasal nitric oxide; SI, situs inversus; SS, situs solitus; FEV1, forced expiratory volume in 1 s. ^a^ PCD specific cut-off of < 77 nL/min; ^b^ Pathogens: Ps a, Pseudomonas aeruginosa; St p, Streptococcus pneumoniae, Kl p Klebsiella pneumoniae, N/A = not available; ^c^ Ashkenazi Jewish; ^d^ At age 16/34 yrs; ^e^ At age 34 yrs; ^f^ Splice-site mutations interrogated with RT-PCR; ^g^ Two transcripts were observed predicting two different translation products.

**Table 2:** Quantification of ODA in subjects with mutations in *ODAD1*.

Subject #	Genotype	# of Cilia Examined	# of Cilia w ODA (%)	# of ODA	# of ODA/# Cilia with ODA
78	c.1502+5G>A c.1502+5G>A	63	18 (28)	92	5.1
897	c.1502+5G>A c.853G>A	43	12 (28)	65	5.4
359	c.853G>A c.853G>A	36	4 (11)	18	4.5
360	c.853G>A c.853G>A	35	5 (14)	8	1.6
891	c.853G>A c.598-2A>G	55	0	0	0
961	p.Arg150* p.Arg150*	34	0	0	0
1107	c.853G>A c.1050delT	53	0	0	0

## Data Availability

The data presented in this study are available on reasonable request from the corresponding author.

## Supplementary Materials

Supporting information, including 3 figures, 3 Tables, and 4 videos, can be downloaded at: https://www.mdpi.com/article/10.3390/ijms23031753/s1.
