# Supplementary material for: Expression of a Truncated Form of ODAD1 Associated with an Unusually Mild Primary Ciliary Dyskinesia Phenotype
Source: Int J Mol Sci. 2022 Feb 3;23(3):1753. doi: 10.3390/ijms23031753 (PMC8835943; doi:10.3390/ijms23031753)
Supplement: Supplementary file 1 [file ijms-23-01753-s001.zip › ijms-1543469 - REVISED Supplementary w Fig S3.pdf]

Supplementary Materials

Figure S1. Ciliary waveform analysis of cultured cells from UNC78 and UNC77

Figure S2. Characterization of ODAD1 antisera against in vitro expressed cDNAs

Figure S.3 Detection of DNAI1 protein in cilia from UNC78

Table S1. Primers used for PCR studies

Table S2. Primers and probes used for ddPCR

Table S3. Antibodies used in this study

Video S1. Video showing ciliary activity in control HNE cell culture.

Video S2. Video showing ciliary activity in UNC78 cell culture.

Video S3. Video showing ciliary waveform in UNC77 cell culture.

Video S4. Video showing ciliary waveform in UNC78 cell culture.

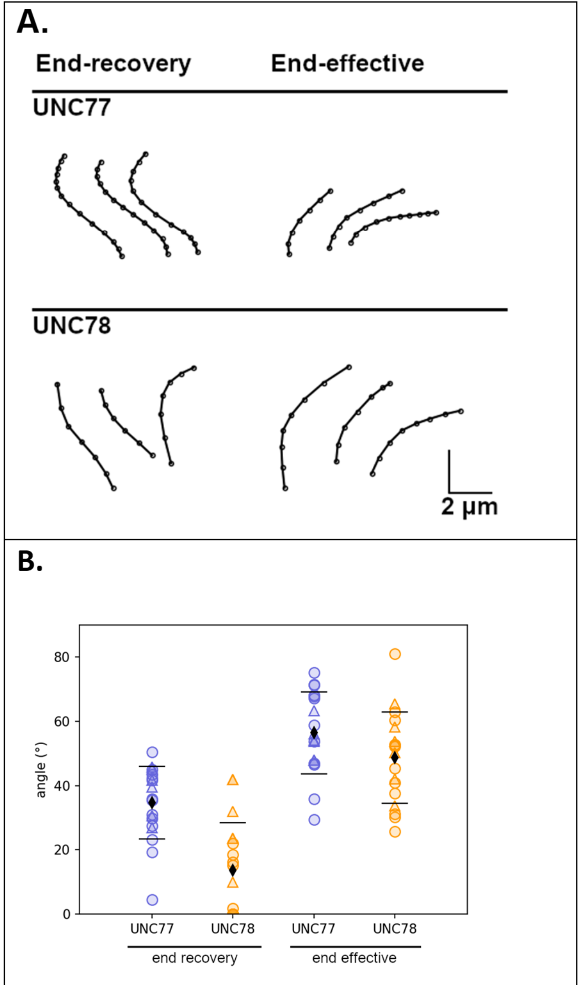

Figure S1. Waveform analysis of UNC78 appears to show an altered recovery stroke. A) Cilia were traced in profile view at two time points in the cycle, the end recovery position and end effective position. Each cilium was traced by marking positions along the cilium on a video frame. The vertical direction for a cilium was defined by the first (most proximal) segment. The angle measured was the greatest angle any segment made with vertical. This angle was measured in the recovery direction for the end recovery position and in the effective direction for the end effective position in two experiments. B) Each graph symbol shows the angle for one cilium (first experiment, triangles; second experiment, circles). The samples are either UNC77 (blue; end recover n_cilia=19, end effective n_cilia=18) or UNC78 (orange; end recover n_cilia=18, end effective n_cilia=18). UNC77 is a parent of UNC78; obligate heterozygote for c.1502+5G>A. In the first experiment, cilia came from two UNC77 and two UNC78 cells, and in the second experiment from four UNC77 and four UNC78 cells. In all but one case, three cilia were measured per cell. The overall mean (black diamond) and standard deviations (black horizontal bars) are shown. Differences between UNC77 and UNC78 were not significant.

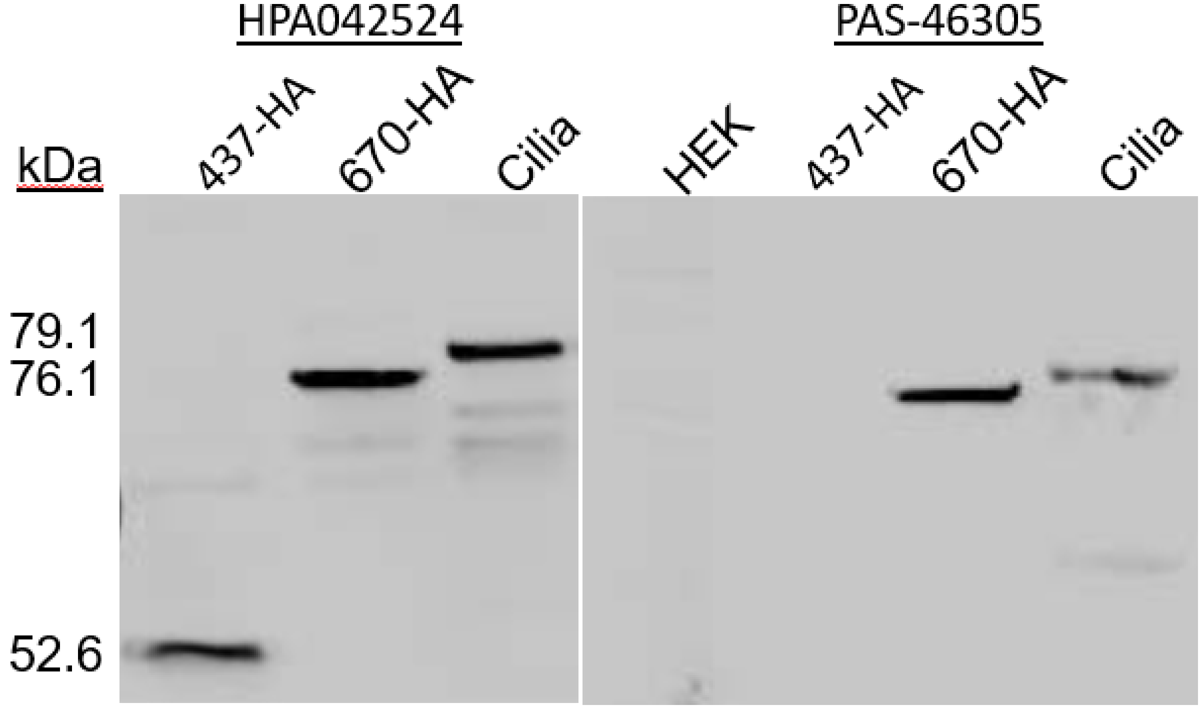

Figure S2. Characterization of ODAD1 antisera. Total proteins were isolated from HEK cells expressing HA tagged cDNAs of the 670 and 437 amino acid isoforms of ODAD1 and from control HBE ciliary axonemes. Western blots were probed with antisera raised against the mid-region (42524) or carboxy terminal region (PAS-46305) of ODAD1. Both antisera react with the full-length versions of ODAD1, while only 42524 recognizes the truncated protein. Note that the ciliary protein is slightly larger than the expressed 670 aa isoform due to an additional 37 aa at the amino terminus.

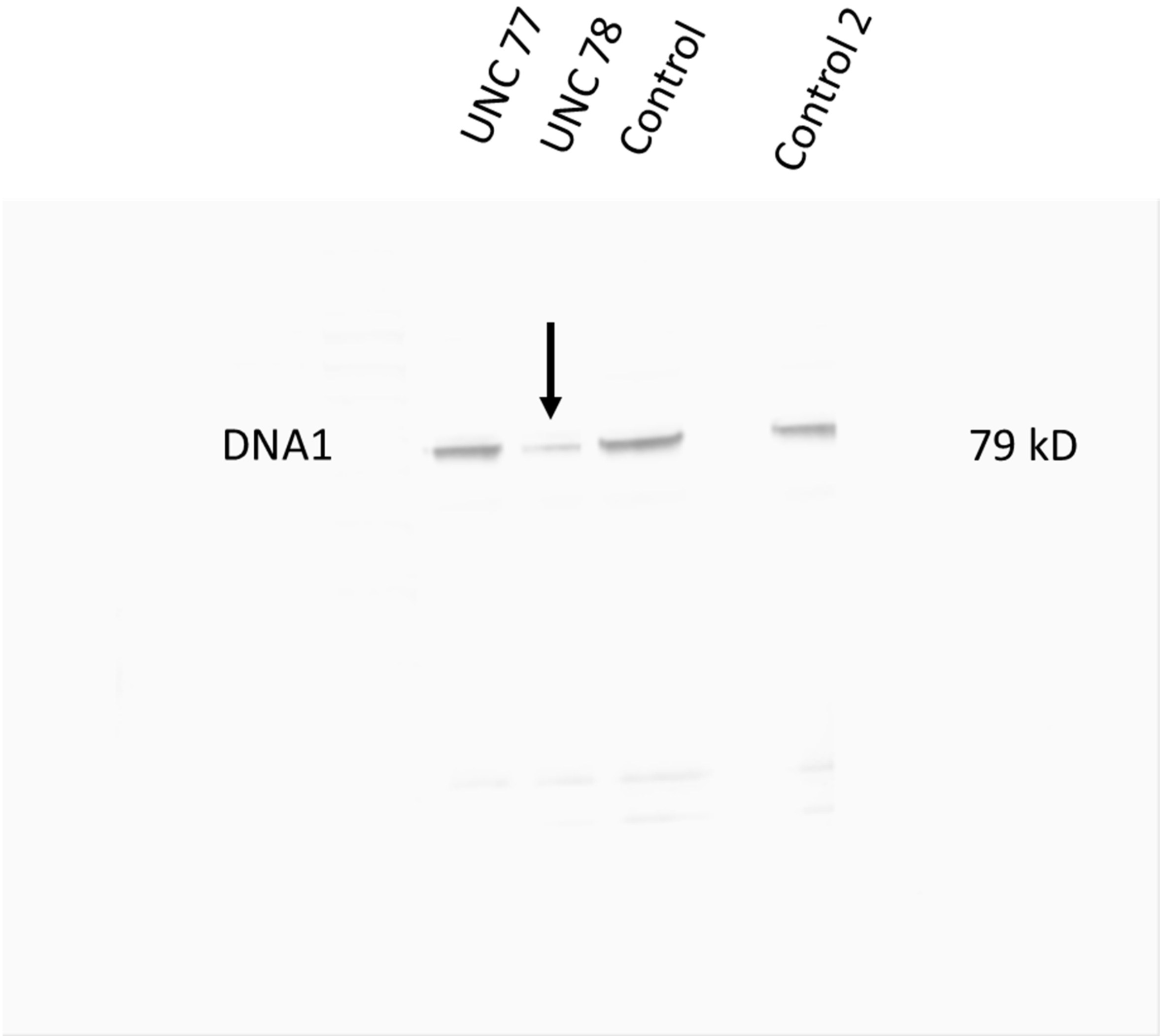

Figure S3. Detection of DNAI1 protein in cilia from UNC78. Isolated cilia from differentiated cultures of UNC77, UNC78, and two control HBE cultures were analyzed by Western blotting and probing with an antibody to the axonemal outer dynein arm protein, DNAI1. The presence of a signal for DNAI1 (black arrow) in the UNC78 sample provides additional evidence that some axonemal outer dynein arms are assemble. Entire original blot is shown (n=2).

Table S1 Primers used for RT-PCR				
**Gene**	**Primer Sequence**	**Exon**	**PCR product**	**Annealing**
ODAD1-F	GCCAGCAAGGATGACCAGCATT	12	478 bp	63°C
ODAD1-R	TTCTCCACTTGGCTCAGCAGCT	15	478 bp	63°C

Table S2 Primers used for ddPCR			
**Primer Name**	**Sequence (5' – 3')**	**Amplicon Length (bp)**	**Annealing Temperature (°C)**
***ODAD1* Exon 10 - 11**		172	60
Ex. 10_F	GAGGCTGGTGCTTTGCTAC		
Internal Oligo	AGGACGCCCTGAATAAACTGTCCCAGCTGA		
Ex. 11_R	GATCTCTTCCTGCACATGCT		
***ODAD1* Exon 13 - 14**		133	60
Ex. 13_F	CCTGAGCCTCATTGAGAAGC		
Internal Oligo	TCCCTGGCCGACGCTGCCCT		
Ex. 14_R	CTTCTTCGGAAGGTCCTCCA		
***DNAI1* Exon 3 – 5 (BioRad Assay ID: dHsaCPE5035646)[a]**		71	60
	CCCTGACCAGCTGGAGTTGACCGATGCGGAG TTAAAGGAGGAGTTCACTCGGATTTTGACAGC CAACAACCCACACGCACCCCAGAACATTGTCA GGTACAGCTTCAAAGAAGGCACATATAA		

[a]ddPCR gene expression probe assays were acquired from BioRad. Sequences of the primers and fluorescent internal oligonucleotide are proprietary, so the region containing the amplicon sequence is provided instead.

Table S3 Antibodies used in this study					
**Antibody Name**	**Host**	**Company**	**Catalog #**	**Dilutions WB**	**Dilutions IF**
CCDC114 (ODAD1-M)	Rabbit	Atlas	HPA042524	1:20,000	1:500
CCDC114 (ODAD1-C)	Rabbit	Invitrogen	PA5-46305	1:3000	1:250
Acetylated Tubulin	Mouse	Sigma	T7451	NA	1:500
HA-Tag	Rabbit	Sigma	H 6908	1:4000	NA
RSPH 1A	Rabbit	Novusbio	NBP1-89839	1:4000	NA
IRDye 800CW secondary	Donkey	Licor	926-32213	1:15,000	NA
IRDye 680CW secondary	Donkey	Licor	925-68073	1:15,000	NA
Anti-Mouse Alexa Fluor 488	Donkey	Invitrogen	A-21202	NA	1:500
Anti-Rabbit Rhodamine Red-X	Donkey	Jackson ImmunoResearch	711296152	NA	1:500
